# Contributions from incumbent police officer’s physical activity and body composition to occupational assessment performance

**DOI:** 10.3389/fpubh.2023.1217187

**Published:** 2023-06-21

**Authors:** Nathan D. Dicks, Marni E. Shoemaker, Kathryn J. DeShaw, Michael J. Carper, Kyle J. Hackney, Allison M. Barry

**Affiliations:** ^1^Department of Health, Nutrition, and Exercise Sciences, North Dakota State University, Fargo, ND, United States; ^2^School of Health and Consumer Sciences, South Dakota State University, Brookings, SD, United States; ^3^Kinesiology Program, Loras College, Dubuque, IA, United States; ^4^Department of Health, Human Performance, and Recreation, Pittsburg State University, Pittsburg, KS, United States

**Keywords:** law enforcement, body fat percentage (BF%), cardiovascular fitness, physical readiness assessment, leisure time activities

## Abstract

**Introduction:**

Police officers must perform various tasks in unpredictable work environments and potentially volatile situations. This study aimed to determine if cardiovascular fitness, body composition, and physical activity levels could predict performance in a Midwest Police Department’s Physical Readiness Assessment (PRA).

**Methods:**

Researchers collected data from thirty incumbent police officers (33.9 ± 8.3 years, female = 5). Anthropometric data included height, body mass, body fat percentage (BF%), fat-free mass (FFM), and maximal hand grip strength. The police officers also completed a physical activity rating (PA-R) scale to estimate maximal oxygen consumption ($\dot{V}$ O_2max_) and the International Physical Activity Questionnaire (IPAQ). Police officers then conducted their department’s PRA. Stepwise linear regression analyses were used to determine the relationship between predictor variables and PRA performance. Pearson’s product-moment correlations investigated relationships between anthropometric, physical fitness, and physical activity variables and PRA performance using SPSS (v.28). The significance level was set at *p* < 0.05.

**Results:**

Descriptive data for the sample includes BF%: 27.85 ± 7.57%, FFM: 65.73 ± 10.72 kg, hand grip strength: 55.51 ± 11.07 kg, weekday sedentary time (WST): 328 ± 28.26 min, weekend day sedentary time (WDST): 310 ± 28.92 min, daily moderate-to-vigorous physical activity (MVPA): 29.02 ± 39.41 min, PRA: 273.6 ± 51.4 s and estimated $\dot{V}$ O_2max_: 43.26 ± 6.35 mL kg^−1^ min^−1^. The stepwise regression analyses indicated that BF% was predictive of PRA time (*R*^2^ = 0.32, *p* < 0.01); estimated $\dot{V}$O_2max_ predictive of PRA time (*R*^2^ = 0.45, *p* < 0.001). There were significant correlations between BF % and PRA time (*r* = 0.57, *p* < 0.001), PA-R and MVPA (*r* = 0.71, *p* < 0.001), %BF % and WDST (*r* = −0.606, *p* < 0.001), hand grip and FFM (*r* = 0.602, *p* < 0.001) and PA-R and PRA time (*r* = −0.36, *p* < 0.05).

**Discussion:**

The results of this exploratory study highlight that higher estimated $\dot{V}$O_2max_ and lower BF% were the best predictors for faster PRA completion times, accounting for 45% and 32% of the variance, respectively. The findings of this study support the need for wellness and fitness initiatives in law enforcement agencies focused on increasing cardiovascular fitness and physical activity while decreasing BF% to ensure optimal performance in policing and overall health.

## 1. Introduction

Police officers are critical in protecting and serving our communities. They are subjected to unique demands while performing various tasks (e.g., running, jumping, pulling, pushing, and carrying) in unpredictable work environments and potentially volatile situations (1–3). Police officers may quickly shift from an inactive state to maximal physical exertion (i.e., from seated in their car to a foot pursuit) (4, 5), all the while performing these activities under load (6, 7). Thus, physical fitness is crucial for the demands of law enforcement (8). However, during most shifts, they primarily engage in sedentary behavior, such as sitting in a patrol car for extended periods, sitting in meetings, or doing administrative tasks (2). Due to extended inactive periods, followed by acute heavy physical exertion (i.e., high-speed driving, body drags, obstacle clearance, and subject apprehension), there is increased risk for sudden cardiac death. This enhanced strain on the cardiovascular system is even more detrimental for police officers with lower fitness levels (1, 2, 9).

Police officers are at an increased risk of obesity, hypertension, high cholesterol, diabetes, and cardiovascular disease (CVD) than the general population (4, 10–12). On average, 40% of officers are obese, about 5% higher than the national average, 74% classify with elevated-to-stage two hypertension, and 27% have three or more CVD risk factors (3, 13). More specifically, 83% of officers in the Midwest were considered overweight or obese, with similar findings in retired officers (4). High sedentary time, physical inactivity, and poor dietary habits exacerbate the prevalence of obesity in law enforcement (3, 13, 14). In a study by Vuković et al. (15), researchers found that frequency and volume of leisure time physical activity (LTPA) correlated with lower levels of body fat percentage (BF%) (*r* = −0.306, *p* ≤ 0.001 and *r* = −0.370, *p* ≤ 0.001) respectively. The researchers found that the ideal volume of 150–300 min per week was associated with reduced BF% (15). Additionally, Kukić et al. (16) found that 73.3% of the sixty general police officers were categorized as sedentary with ≤149 min of physical activity (PA) per week, and only 21.7% were considered moderately active with 150–300 min of PA per week. Concurrently, higher levels of obesity correlate with reduced levels of overall fitness (14) and increased risk for cardiometabolic disease (17). Body composition assessment can help recognize and monitor the effects that PA and dietary habits have on police officers (16), which can be done alongside a physical fitness assessment.

Different law enforcement agencies utilize a variety of tests of physical ability or fitness standards. Some of these testing procedures include an occupational readiness assessment that incorporates climbing stairs, jumping, crawling, and changes in direction, or the Cooper standard test (e.g., push-ups, sit-ups, vertical jump, 300 m run, and 2.4 km run). Although no universally accepted physical fitness standards exist for incumbent officers, departments commonly use an occupational readiness assessment to assess physical fitness and fit for duty when returning from injury (18–20). Additionally, a multitude of assessments have been associated with different performance and body composition measurements, indicating the importance of finding main factors influencing testing performance. In a study by Rhodes et al. (21), 55% of the variance, when completing a physical abilities test, was accounted for by maximal aerobic power and anaerobic capacity with a sample mean $\dot{V}$O_2max_ and BF% of 42.6 mL kg min^−1^ and 22.9%, respectively. Similarly, Beck et al. (18) found aerobic endurance and agility performance contributed to physical ability test times. The researchers also found that body mass (BM) and abdominal circumference were related to higher completion times. Dawes et al. (8) found the multi-stage fitness test, dominant hand grip strength, and body mass index (BMI) had significant relationships during the performance of a physical abilities test with highway patrol officers. Together, these studies indicate that factors related to body composition and cardiorespiratory fitness may be influential to on-duty task of law enforcement officers.

Collectively, reduced cardiovascular fitness, increased obesity, and physical inactivity are critically essential aspects of occupational health, given body composition and PA level contribute to occupational-specific tasks. This study aimed to determine if cardiovascular fitness, body composition, and physical activity levels could predict performance in a Midwest Police Department’s Physical Readiness Assessment (PRA). We hypothesized that police officers with higher levels of cardiovascular fitness, less sedentary time, greater moderate-to-vigorous physical activity, and lower BF% would perform better on the PRA.

## 2. Materials and methods

### 2.1. Study design and subjects

This study used a cross-sectional design to describe the anthropometric, body composition, self-reported PA level, and hand grip strength measures of incumbent Midwest police officers and identify characteristics correlated with their PRA. A convenience sample of thirty incumbent police officers ages 23–53 years were recruited to participate in this study. The University’s Institutional Review Board approved all the study’s procedures prior to any data collection. All subjects provided written informed consent before enrollment, and each subject completed a medical history form. The exclusion criteria for the study were any known or major signs of cardiovascular, pulmonary, or metabolic disease. Researchers utilized one visit to collect all data from the police officers. Before arrival, the researchers asked the subjects to wear their workout clothes (e.g., t-shirts, shorts). Researchers collected demographic information, including age, sex, years as a police officer, and physical activity-rating (PA-R) scale (22) through a self-reported survey. The subjects’ anthropometric tests were collected upon completing the self-report survey to confirm participation eligibility in the study. The subjects then completed a physical activity questionnaire and were assessed for body composition and grip strength. Lastly, the officers complete the physical readiness assessment.

### 2.2. Anthropometrics, body composition, and grip strength

Subjects removed any outer garments, shoes, and socks before measuring the height to the nearest 0.5 cm using a stadiometer (Seca, Model 213, Chino, CA, United States). A portable bioelectric impedance analysis (BIA) (Tanita, TBF-300A, Tokyo, Japan) was used to assess BM (kg), BF%, fat mass (kg), and fat-free mass (kg). BIA has been shown to be valid (*r* = 0.934) and reliable (interclass correlation = 0.974–0.994) for body composition assessments in healthy adult populations (23).

A hand dynamometer (Jamar, Lafayette Instruments, Lafayette, IN, United States) was used to assess hand grip strength. The Jamar dynamometer was set at the second handle position to evaluate each subject’s grip strength in kg. The subjects were instructed to stand erect with the shoulder adducted and neutrally rotated, elbow flexed at a 90° angle, forearm in a neutral position, and wrist in slight extension. Subjects were given a countdown and then instructed to squeeze the handle as hard as possible. Subjects were given three attempts on their dominant hand with 30 s rest between trials. The average between the three trials was used for analysis.

### 2.3. Leisure-time physical activity

The International Physical Activity Questionnaire-Long Form (IPAQ) was administered to the subjects to estimate PA in the prior week. The IPAQ consists of four PA domains: household, transportation, work, and aerobic leisure time. The IPAQ is a popular tool to self-report PA and has been shown to have good validity with a median rho of about 0.30 (24). Each domain has a series of prompts to report the number of days per week and time per day spent walking, engaging in moderate or vigorous physical activity (MVPA) intensity were totaled and calculated per day. For the analyses, only the leisure MVPA domain was considered as this is a more relevant factor contributing to overall on-duty performance. The total amount of sitting time was used for calculating weekday sedentary (WST) and weekend day sedentary time (WDST). Subjects were instructed to use their on-duty days as weekdays and off-duty days as weekend days due to the nature of shift work.

### 2.4. Estimated aerobic capacity

The researchers asked subjects to select from the physical activity-rating (PA-R) scale from 0–15. The PA-R scale derived by George et al. (25) accommodates a more robust population concerning PA level (22). Inputs for the predicted $\dot{V}$O_2max_ equation included a self-reported PA-R scale from 0 to 15, age in years, BMI, and sex (1 = male, 0 = female) (26).

$$\begin{array}{c} \mathrm{Estimated}\;\dot{V}O_{2\max}\;\left({\mathrm{mL\cdot kg}}^{-1}\cdot \min^{-1}\right)=56.363+1.921\;\left(\mathrm{PA}-R\right)\\ -0.381\;\left(\mathrm{age}\right)-0.754\;\left(\mathrm{BMI}\right)\\ +10.987\;\left(\mathrm{sex}\right) \end{array}$$

Previous research has reported a strong correlation between the predicted $\dot{V}$O_2max_ estimation equation and a measured graded exercise test (*r* = 0.80 and SEE = 5.36 mL kg^−1^ min^−1^) (27). Data reported by Dicks et al. (28), in firefighters, demonstrated a 7.6% error when comparing the differences between the firefighter’s perceived PA-R to $\dot{V}$O_2max_. Therefore, estimating aerobic capacity using the above estimation equation can be used as a subjective, cost-effective method to assess cardiovascular fitness.

### 2.5. Physical readiness assessment

The PRA consists of several job-related tasks that test the officers’ agility and coordination, aerobic/anaerobic power, muscular strength, and endurance. Officers completed the PRA in athletic clothing. The officers conducted a walk-through familiarization trial to clarify all tasks to be completed. The pass-fail cut-off time was set at 4:40 (minutes: seconds). Briefly, officers completed a six-lap mobility run (390 m total) which included a stair climb simulator, jump obstacle, crawl obstacle, barrier jump, and wall vault. Immediately following the completion of the mobility run, and without rest, the officers transitioned to the push-pull machine, loaded with 36.4 kg, which requires a push phase and a pull phase to simulate the arrest and control of a non-compliant or resistant subject. Between the push and pull phase, the officer executed front and back falls to the floor, simulating being knocked down and recovering to their feet. Once the pull phase was completed, the timer was stopped. After a 60 s rest, the officer was instructed to drag a dummy (45 kg), in a controlled manner, for 15 m. This was an untimed maneuver. The time to complete the PRA was used for analysis.

### 2.6. Statistical analyses

Demographic and anthropometric measures were described using means and standard deviations. The normality of data was confirmed using the Shapiro–Wilk test prior to conducting further statistical analysis. An outlier analysis was conducted to identify variables that were outside of 2 SD from the mean. Three values for MVPA were considered outliers, so the following analyses were performed with and without these subjects (*n* = 3 males). Pearson’s Product–Moment Correlations investigated relationships between anthropometric, physical fitness, and PA variables. The scale for interpretation was as follows: 0.1–0.39 = small, 0.4–0.69 = moderate, and 0.7–0.89 = strong. Stepwise linear regression analyses were used to determine the relationship between selected predictor variables (age, FFM, BF%, PA-R, hand grip strength, WST, WDST, and MVPA) and which variables predicted PRA performance. An Independent samples t-test was used to compare demographic data between the sexes. An additional independent samples *t*-test was used to assess differences in police officers who completed the PRA within the recommended time compared to those who did not. The significance level was set at *p* < 0.05 for all statistical analyses. All analyses were performed using the Statistical Package for the Social Sciences (version 28; SPSS, Inc. Chicago, IL, United States).

## 3. Results

**Figure 1:**
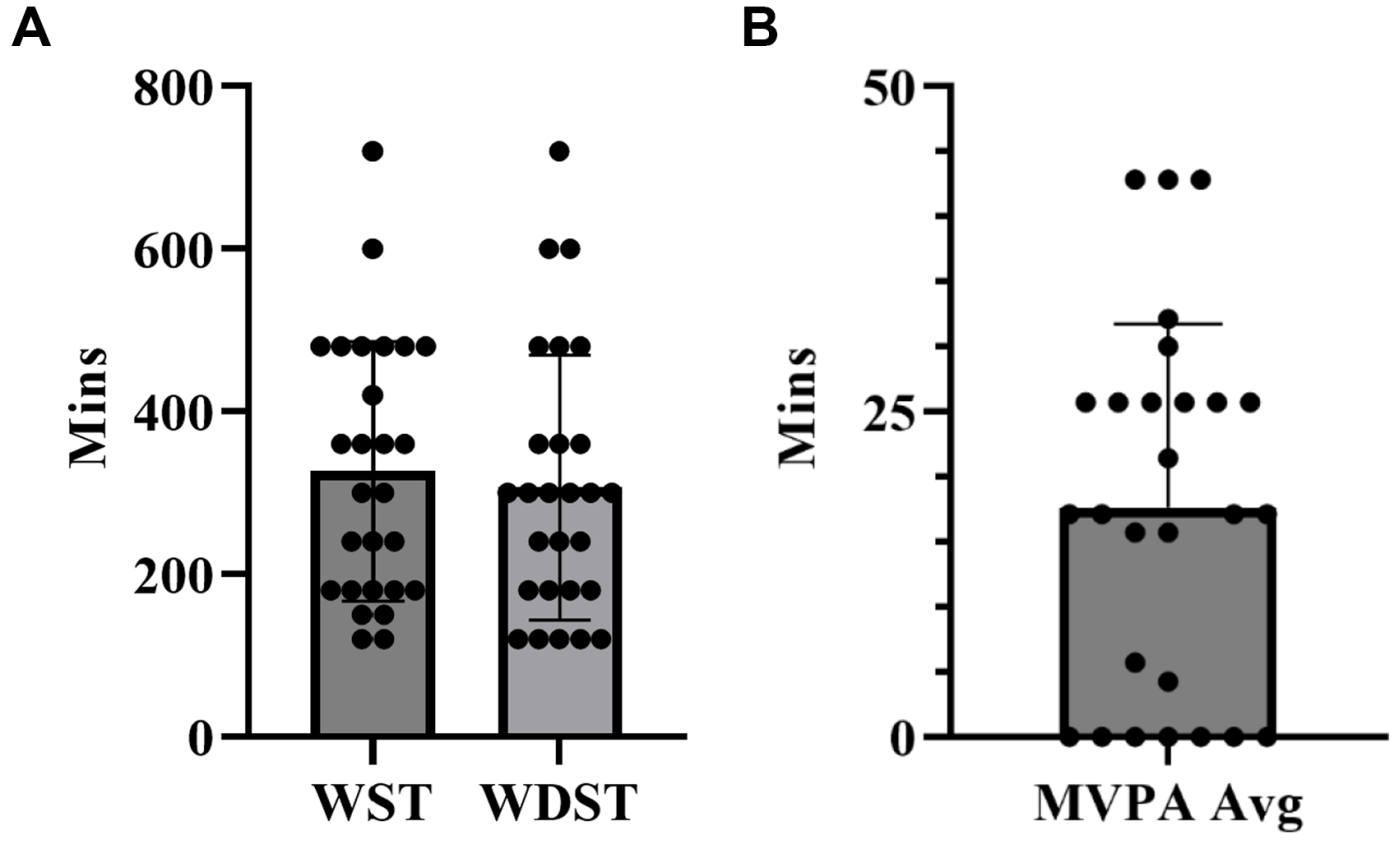
Sedentary time and leisure MVPA by officer. **(A)** Sedentary time comparison between weekday sedentary time (WST) and weekend day sedentary time (WDST) (*n* = 27) with outliers removed. **(B)** By Officer reported leisure moderate to vigorous physical activity (MVPA) (*n* = 27) with outliers removed.

Thirty incumbent police officers (33.9 ± 8.3 years; female = 5) completed the study. These police officers had various duties in the department, which included: uniformed patrol (*n* = 15), school resource officers (*n* = 1), special weapons and tactics (*n* = 1), drug/gang units (*n* = 4), detective (plain clothed) (*n* = 4), community policing (*n* = 4), and administration (*n* = 1). T-tests reported significant differences in height, body mass, fat-free mass (FFM), and hand grip strength between females and males for the total sample and when outliers were removed (*p* < 0.05). It is important to note that when outliers were removed there was a significant difference in PA-R between males and females. T-tests reported significant differences in PA-R, MVPA and estimated $\dot{V}$O_2max_ based on PRA completion time (Table 1). When the outliers were removed MVPA was no longer significant. PA data is described in Figure 1. Using the World Health Organization BMI categories, three officers (10%) were classified as normal weight, 15 (50%) were classified as overweight, and 12 (40%) officers as obese. Descriptive data for the sample are included in Table 1.

**Table 1:** Officer demographics.

Variable	Sample (*n* = 30)	Females (*n* = 5)	Males (*n* = 25)	Met PRA (≤280 s) (*n* = 17)	Did not met PRA (>280 s) (*n* = 13)
Age (years)	33.9 ± 8.3	31.4 ± 5.2	34.4 ± 8.8	32.5 ± 7.0	35.6 ± 9.8
Height (cm)	176.8 ± 8.6	165.4 ± 8.5^**^	179.1 ± 6.8	175.2 ± 7.2	178.9 ± 10.1
Mass (kg)	93.0 ± 17.7	73.3 ± 12.2^*^	95.7 ± 16.3	86.8 ± 13.1	98.8 ± 21.0
BMI (kg m^−2^)	29.2 ± 4.0	26.6 ± 2.5	29.8 ± 4.1	28.2 ± 2.7	30.6 ± 5.0
Body fat (%)	27.9 ± 7.6	33.7 ± 5.0	26.7 ± 7.5	25.7 ± 6.4	30.7 ± 8.3
FFM (kg)	65.7 ± 10.7	48.2 ± 5.6^**^	69.2 ± 7.6	64.2 ± 8.6	67.7 ± 13.1
Hand grip strength (kg)	55.5 ± 11.1	41.0 ± 9.6^**^	58.4 ± 9.0	56.4 ± 12.4	54.3 ± 9.4
PA-R (0–15)	5.7 ± 1.6	6.2 ± 0.5^†^	5.6 ± 1.7	6.3 ± 1.3^*^	4.9 ± 1.5
PRA (sec)	273.6 ± 51.4	304.4 ± 45.0	267.4 ± 51.1	236.8 ± 30.2^**^	321.8 ± 26.8
Career (years)	9.2 ± 8.6	7.2 ± 6.5	9.6 ± 9.1	7.6 ± 6.1	11.4 ± 11.0
MVPA (min)	29.0 ± 39.4	30.9 ± 11.5	28.7 ± 43.1	41.5 ± 48.3^*‡^	12.7 ± 11.5
Est $\dot{V}$O_2max_ (mL kg^−1^ min^−1^)	43.3 ± 6.4	37.9 ± 2.2	44.3 ± 6.4	46.5 ± 5.2^**^	39.0 ± 5.3

Mean ± standard deviation, BMI, body mass index; FFM, fat-free mass; PA-R, physical activity-rating; PRA, physical readiness assessment; career, years as police officer; MVPA, moderate to vigorous physical activity; Est $\dot{V}$O_2max_, estimated maximal oxygen consumption. Significant differences between females and males; significant difference between met PRA and did not met PRA. ^*^p < 0.05, ^**^p < 0.001. Significant difference with outliers removed (*n* = 3). ^†^*p* < 0.05, ^‡^no longer significant with outliers removed (*n* = 3). Outliers were all male.

Two linear regression models were used to identify predictors of PRA time to completion. The first model used BF%, FFM, BMI, hand grip strength, years on the job, WST, WDST, and MVPA as independent variables. This model demonstrated that 32% of the variance in PRA time could be predicted by BF% [*F* (1, 29) = 13.31, *R*^2^ = 0.32, *p* < 0.01] (Table 2). Comparatively, when outliers were removed, 26% of the variance in PRA time could be predicted by BF% [*F* (1, 26) = 8.881, *R*^2^ = 0.262, *p* < 0.01] (Table 2). When estimated $\dot{V}$O_2max_ was added as an independent variable to the regression model (Table 3), only estimated $\dot{V}$O_2max_ was predictive of PRA time to completion [*F* (1, 29) = 22.84, *R*^2^ = 0.45, *p* < 0.001]. Additionally, only estimated$\dot{\;V}$O_2max_ was predictive of PRA time to completion, contributing 37% of the variance to the model when outliers were removed [*F* (1, 26) = 14.829, *R*^2^ = 0.37, *p* < 0.001].

**Table 2:** Stepwise regression model.

Variable	*B*	SEB	*β*
Total sample constant	166.402^***^	30.416	
BF%	3.849^**^	1.055	0.568
Outliers removed constant	181.076^***^	34.057	
BF%	3.440^**^	1.154	0.512

SEB, standard error for the unstandardized beta. ^*^p < 0.05, ^**^p < 0.01, ^***^p < 0.001.

**Table 3:** Stepwise regression model.

Variable	*B*	SEB	*β*
Total sample constant	508.098^***^	49.574	
Est $\dot{V}$O_2max_	−5.421^***^	1.134	−0.670
Outliers removed constant	502.65^***^	58.522	
Est $\dot{V}$O_2max_	−5.285^***^	1.372	−0.610

SEB, standard error for the unstandardized beta. ^*^p < 0.05, ^**^p < 0.01, ^***^p < 0.001.

There were significant correlations between estimated BF% and PRA time (*r* = 0.57, p < 0.001), PA-R and MVPA (*r* = 0.71, p < 0.001), BF % and WDST (*r* = −0.61, p < 0.001), hand grip and FFM (*r* = 0.60, p < 0.001) and PA-R and PRA time (*r* = −0.36, *p* < 0.05). When the three outliers were removed from the sample, BM and PA-R became significant (*r* = −0.38, p < 0.05). However, both age and estimated $\dot{V}$O_2max_ (*r* = −0.35, *p* = 0.08) and MVPA and estimated $\dot{V}$O_2max_ (*r* = 0.20, *p* = 0.330) was nonsignificant. Table 4 reports additional correlations between anthropometric, physical fitness, and PA variables.

**Table 4:** Anthropometric, physical fitness, and physical activity variables.

Variable	1	2	3	4	5	6	7	8	9	10	11	12
1. Age	—	−0.01	0.22	0.03	0.34	0.11	−0.01	0.38^*^	−0.27	−0.38^*^	−0.56	−0.37^*‡^
2. Height		—	0.73^***^	0.86^**^	−0.06	−0.41^*^	−0.23	−0.01	−0.01	0.19	−0.10	−0.09
3. Body mass			—	0.78^***^	0.48^**^	0.44^*^	−0.34^†^	0.29	−0.18	−0.14	−0.11	−0.32^*^
4. Fat-free mass				—	−0.15	0.60^***^	−0.18	−0.05	0.06	0.25	0.03	0.13
5. Body fat %					—	−0.12	−0.23	−0.57^***^	−0.36^*^	−0.61^***^	−0.20	−0.72^***^
6. Hand grip						—	0.07	−0.20	−0.07	0.01	0.08	0.16
7. PA-R							—	−0.36^*^	0.07	0.07	0.71^***^	0.56^***^
8. PRA								—	−0.04	−0.21	−0.34	−0.67^***^
9. WST									—	0.60^***^	0.10	0.27
10. WDST										—	0.09	0.46^**^
11. Leisure MVPA											—	0.49^**‡^
12. Est $\dot{V}$O_2max_												—

PA-R, physical activity-rating; PRA, physical readiness assessment; WST, weekday sedentary time; WDST: weekend day sedentary time; MVPA, moderate-vigorous physical activity. ^*^p < 0.05, ^**^p < 0.01, ^***^p < 0.001. Significant difference with outliers removed (*n* = 27) ^†^*p* < 0.05, ^‡^no longer significant with outliers removed (*n* = 27).

## 4. Discussion

The current study sought to determine if cardiovascular fitness, body composition, and PA levels could be used as predictors of PRA time to completion in Midwest police officers. Our data demonstrates that BF% and estimated $\dot{V}$O_2max_ were predictors of PRA time. These findings are similar to previous research (8, 18), indicating that individual officers who are more fit and who possess a lower BF% may perform better on completing an occupational physical abilities assessment.

The current department utilized a recommended PRA time to completion standard of 4:40 (minutes: seconds) or 280 s. As a result, 17 officers completed the PRA, and 13 did not meet the PRA in the allotted time. These results agree with Dawes et al. (8), where high and low performers were identified based on completion or non-completion of various physical abilities and multi-stage fitness tests. It should be noted that the officers in the current study did complete the assessment in gym clothes compared to their duty gear, which the authors suspect the times would have been slower with the addition of body armor, duty belt, uniform, and boots.

The officers who completed the PRA in the allotted time (*n* = 17) were estimated to have a $\dot{V}$O_2max_ of 46.5 ± 5.2 mL kg^−1^ min^−1^, compared with those that did not complete the PRA in the allotted time ($\dot{V}$O_2max_ of 39.0 ± 5.3 mL kg^−1^ min^−1^). In comparison, current $\dot{V}$O_2max_ recommendations for firefighters is 42 mL kg^−1^ min^−1^ based on the anticipated job tasks, while dressed in personal protective equipment and exposed to various environmental stressors (29). Based on the similarities police officers share in occupational demands to firefighters, sharing this standard between police officers and firefighters is supported (30). Thus, there should be a recommended minimal $\dot{V}$O_2max_ measure for police officers.

Even with a 7.6% error reported with subjective PA-R (28), the recommendation of 42 mL kg^−1^ min^−1^ significantly determines officers meeting the standard in the PRA in this sample (*p* = 0.002). Assessing cardiovascular fitness in police officers is commonly completed through the 2.4 km run test or the 20-meter multi-stage fitness test (31). The results from these fitness tests can be utilized in formulas to estimate $\dot{V}$O_2max_ (32, 33). Should these methods continue to be used to assess aerobic and work capacities, they should also be used to assist in creating a fitness plan to strengthen the cardiovascular health of these officers. If law enforcement departments choose only to use an occupational readiness assessment to measure overall fitness, the time to completion standards should evoke a similar $\dot{V}$O_2_ response with the recommended standard of 42 mL kg^−1^ min^−1^.

This exploratory study highlights that lower BF% was the best predictor for faster PRA completion times, where BF% accounted for 32% of the variance. Additionally, officers that met the PRA time standard had significantly less BF% than those who were unable to complete the PRA in the allotted time. Prior research has found that BF% negatively correlated with vertical jump performance and estimated oxygen consumption and positively correlated with 2.4 km run time (34). Another study reported decreases in vertical jump performance and stride velocity in law enforcement recruits due to a 9 kg increase in load carriage weight (considered non-FFM) (35). Furthermore, with part-time special weapons and tactics officers, higher BF% resulted in slower obstacle course times (36). Thus, our data support the literature that a high %BF is associated with slower PRA completion times.

These officers’ decreased WDST and hand grip strength levels were associated with lower BF% and higher FFM, respectively. Thus, the officers with higher PA and greater muscle strength experienced lower BF%. Therefore, exercise programs for officers should focus on developing muscular strength and aerobic training. Previous research found that the ideal volume of 150–300 min per week of PA was associated with reduced BF% (15). Without any PA recommendations for officers, considerations should accommodate the physical inactivity that can be prevalent during their shifts. The current study highlights the need to focus on initiatives that may decrease BF.

In this sample, only five police officers reported 30 min or more of daily leisure MVPA. Three police officers were removed as outliers (with >100 min of MVPA), lowering the average to 17.6 ± 14.1 min. Nine officers reported engaging in ten or fewer minutes of daily leisure MVPA. Similarly, 73.3% of police officers categorized with the IPAQ were considered sedentary with ≤149 min of total MVPA per week (16). However, it is essential to note that subjective assessments tend to conform to the social desirability bias (37). This might be even more pronounced in the sedentary time as the officers had roughly 5–7 h per day of sedentary time during the weekday and weekend days, which could be relatively low with the sedentary nature of the job.

The results of the current study indicate that officers should focus on improving $\dot{V}$O_2max_, BF%, and MVPA to shorten their PRA completion times and to improve overall physical readiness to perform on-duty tasks (e.g., running, pulling, pushing, lifting, and carrying). Appropriately structured wellness initiatives within the law enforcement community should target improvement in these outcomes to ensure officers possess a certain level of cardiovascular fitness to perform their duties safely and to reduce the risk of cardiovascular disease development.

### 4.1. Strengths and limitations of the study

This study has several strengths. First, the study used a sample of incumbent police officers to assess cardiovascular fitness, body composition, and physical activity levels to complete a PRA. Second, the use of a PA assessment is due to a lack of evidence in the literature citing PA levels for incumbent officers. Third, using a time-dependent PRA to identify what attributes make an officer successful in completing the PRA within the allotted time. The limitations of this study include the estimation of $\dot{V}$O_2max_ using a non-exercise equation and the measurement of PA using a subjective survey (IPAQ). The self-reported measures lead to the misclassification of PA intensities, thus, creating the need to control for outliers to represent the whole sample better.

## 5. Future research

The findings of this study provided more understanding of the roles of cardiovascular fitness, body composition, and PA in improving occupational readiness assessments. Officers have demanding occupations and should maintain adequate levels of physical fitness as a part of their job performance and health outcomes. Future research should include a graded exercise test with measured $\dot{V}$O_2max_, for cardiorespiratory fitness. This can be in addition to the $\dot{V}$O_2max_ estimations from completion of the 2.4 km run or the 20 meter multi-stage fitness test. The data supports the need for wellness and fitness initiatives in law enforcement agencies focused on increasing cardiovascular fitness and decreasing BF% while increasing PA to ensure optimal performance in policing duties and overall health. Future research should include modification of the factors outlined in this study to improve officer-focused occupational readiness assessments, identify approaches/methods for decreasing periods of physical inactivity while on-duty, and engage in more PA for officers off-duty.

## Data availability statement

The raw data supporting the conclusions of this article will be made available by the authors, without undue reservation.

## Ethics statement

The studies involving human participants were reviewed and approved by North Dakota State University Institutional Review Board. The patients/participants provided their written informed consent to participate in this study.

## Author contributions

ND, AB, and KH contributed to the conception and design of the study and contributed to the visualization. ND performed the investigation. ND, KD, AB, and KH performed the statistical analysis. ND, MS, KD, KH, and AB contributed to interpreting the data. ND and AB wrote the first draft of the manuscript. MS, KD, and MC wrote sections of the manuscript. All authors contributed to the article and approved the submitted version.

## Conflict of interest

The authors declare that the research was conducted in the absence of any commercial or financial relationships that could be construed as a potential conflict of interest.

## Publisher’s note

All claims expressed in this article are solely those of the authors and do not necessarily represent those of their affiliated organizations, or those of the publisher, the editors and the reviewers. Any product that may be evaluated in this article, or claim that may be made by its manufacturer, is not guaranteed or endorsed by the publisher.
